# Severe chronic kidney disease environment reduced calcium-sensing receptor expression in parathyroid glands of adenine-induced rats even without high phosphorus diet

**DOI:** 10.1186/s12882-020-01880-z

**Published:** 2020-06-09

**Authors:** Taketo Uchiyama, Ichiro Ohkido, Akio Nakashima, Yatsumu Saito, Masataka Okabe, Takashi Yokoo

**Affiliations:** 1 Division of Nephrology and Hypertension, Department of Internal Medicine, the Jikei University School of Medicine, Tokyo, Japan; 2 Department of Anatomy, the Jikei University School of Medicine, Tokyo, Japan

**Keywords:** Calcium-sensing receptor, Vitamin D receptor, Glial cells missing 2, CKD-MBD, Secondary hyperparathyroidism

## Abstract

**Background:**

Chronic kidney disease (CKD) disrupts mineral homeostasis and its main underlying cause is secondary hyperparathyroidism (SHPT). We previously reported that calcium-sensing receptor (CaSR) mRNA and protein expression in parathyroid glands (PTGs) significantly decreased in a CKD rat model induced by a 5/6 nephrectomy that were fed a high phosphorus diet. However, there was a significant difference in the severity of CKD between high phosphorus and adequate phosphorus diet groups. Thus, it was unclear whether CKD environment or the high phosphorus diet influenced *CaSR* expression, and the underlying mechanism remains largely unknown.

**Methods:**

CKD was induced in rats with 0.75% adenine-containing diet. CKD and control rats were maintained for 5 days and 2 weeks on diets with 0.7% or 1.3% phosphorus. For gene expression analysis, quantitative real-time polymerase chain reaction was performed with TaqMan probes. Protein expression was analyzed by immunohistochemistry.

**Results:**

PTG CaSR expression significantly decreased in the presence of a severe CKD environment, even without the high phosphate load. Ki67 expressing cells in PTGs were significantly higher only in the CKD rats fed a high phosphorus diet. Furthermore, among the many genes that could affect *CaSR* expression, only vitamin D receptor (VDR) and glial cells missing 2 (Gcm2) showed significant changes. Moreover, *Gcm2* was significantly reduced at an early stage without significant changes in serum calcium, phosphorus and 1,25(OH)_2_ vitamin D, and there was no significant reduction in *CaSR* and *VDR* expressions. Then, significantly elevated Ki67-positive cell numbers were also only observed in the early CKD PTGs with high-phosphorus diets.

**Conclusions:**

Our data suggest that the cause of the decreased PTG *CaSR* expression is the reduction in *VDR* and *Gcm2* expression; Gcm2 may play a role in the onset and progression of SHPT.

## Background

Chronic kidney disease (CKD) is a global epidemic and public health problem, affecting human health in > 10% of the world population [1]. CKD is an independent predictor of all-cause mortality as well as cardiovascular mortality [2]; therefore, it is important in controlling CKD progression. CKD mineral and bone disorder is not only as a result of abnormalities in serum calcium (Ca), phosphorus (Pi), parathyroid hormone (PTH), vitamin D, and fibroblast growth factor 23 (FGF23), rather it is defined as a systemic disorder that is strongly associated with higher rates of all-cause and cardiovascular-related mortalities [3]. Parathyroid glands (PTGs) play a major role in controlling mineral homeostasis, and PTGs change its morphology and function as CKD progresses and causes secondary hyperparathyroidism (SHPT), an important CKD complication. SHPT is strongly correlated with cardiovascular disease-related mortality and morbidity [4], and its pathological elucidation of SHPT is essential. SHPT occurs wherein renal impairment is still fairly early; therefore, it requires early therapeutic intervention. The expression of calcium-sensing receptor (CaSR) in the PTGs directly modulates PTH secretion and probably contributes to parathyroid hyperplasia [5, 6]. In the PTGs, there is a gradual decrease in CaSR expression, which is important in the onset and progression of SHPT, with CKD progression [7], and it is also associated with treatment resistance; however, the mechanism is largely unknown. CaSR mRNA and protein expressions significantly decreased in the rats fed a high phosphorus diet in our CKD rat model with SHPT induced by 5/6 nephrectomy [8]. However, because in the nephrectomized rats, there was a significant difference in CKD severity between the high and adequate phosphorus diet groups, it was unclear which environment, the CKD environment or the high phosphorus diet, had a stronger influence on *CaSR* expression. Therefore, in this study, we focused on the effects of the CKD environment and high phosphorus diet on PTGs and analyzed the mechanism of CaSR decrease in the PTGs. Among rats fed a normal or a high phosphate diet, we induced CKD using adenine. CKD environment indicates that the filtration capacity of the kidney is reduced and the normal state cannot be maintained. In this experiment, the state in which the small molecules, i.e., urea nitrogen (UN) and creatine (Cr), increased was known as CKD. The severity was then expressed by the degree of the value.

The upstream genes directly control the *CaSR* expression, vitamin D receptor (*VDR*), and glial Cells Missing 2 (*Gcm2*) [9, 10]. VDR is a heterodimer with retinoid X receptor α (RXR) to control the transcriptional activity of target genes according to binding to vitamin D response elements [11]. *Gcm2* is essential for parathyroid development in terrestrial vertebrates [12]; furthermore, Gcm2 directly transactivates *CaSR* via binding to Gcm2 response elements in the *CaSR* promoter region [10]. VDR and Gcm2 can cause decreased CaSR expression during SHPT progression. Subsequently, we focused on the transcription factors that directly and indirectly regulate *Gcm2* expression according to the differentiation and proliferation during the development of parathyroid cells. SHPT is the result of excessive differentiation of parathyroid cells, and some developmental genes are utilized for tissue maintenance, such as Pdx1 during pancreatic development [13, 14] and HGF during hepatocyte development [15]. Approximately 10 transcription factors are crucial for parathyroid development, and when their gene function is impaired, parathyroid dose not develop. Although many of these genes do not directly regulate *Gcm2* expression, the upstream and downstream genes of *Gcm2* are inferred depending on the expression time during the developmental stage [16]. Accordingly, we focused on the transcription factors that do not induce *Gcm2* expression and cause aparathyroidism; therefore, it makes sense to focus on the genes such as paired box 1 (*Pax1*), paired box 9 (*Pax9*), sine oculis-related homeobox 1 (*Six1*), sine oculis-related homeobox 4 (*Six4*), eyes absent homolog 1 (*Eya1*), homeobox A3 (*Hoxa3*), forkhead box i1 (*Foxi1*), forkhead box i3 (*Foxi3*), trans-acting T-cell-specific transcription factor GATA-3 (*GATA3*), v-maf musculoaponeuroticfibrosarcoma oncogene homologue B (*MafB*), and t-box transcription factor 1 (*Tbx1*).

We investigated the expression of these genes and demonstrated that severe CKD environment caused the suppression of PTG *CaSR* even in the absence of a high phosphorus diet. Moreover, we verified that only a CKD environment, without phosphorus overload, caused a significant suppression of *VDR* and *Gcm2* similar to CaSR change.

## Methods

### Animals

All animal experiments were performed in accordance with the guidelines of the Animal Care and Use Committee of The Jikei University School of Medicine. The rats were acclimatized to the laboratory conditions for 1 week prior to experimentation. Seven-week-old adult male Wistar rats purchased from CLEA-Japan (Tokyo, Japan) were fed a standard diet (CE-II) with free access to food and water until they were 8-week-old (three rats per cage).

### CKD model and dietary phosphorus intake

CKD was induced by orally feeding rats 0.75% adenine to rats for 5 days and 2 weeks, which were denoted as 5DCKD and CKD groups, respectively. Control groups were prepared using adenine-free diet, denoted as 5Dcontrol and control groups, respectively. Adenine-free and adenine-containing diets were further divided into normal phosphate (NP) containing 0.9% calcium, 0.7% phosphorus, and 1000 IU/kg vitamin D, as well as high phosphate (HP) diet containing 0.9% calcium, 1.3% phosphorus, and 1000 IU/kg vitamin D. Thus, we classified the rats into eight groups: 5Dcontrol NP, 5Dcontrol HP, 5DCKD NP, 5DCKD HP, Control NP, Control HP, CKD NP, and CKD HP groups. Rats were given free access to food and water while receiving the above-mentioned diets. The experimental protocol is shown in Supplemental Fig. S1.

### Parathyroidectomy

All rats were euthanized using 5% isoflurane anesthesia by cardiac puncture. The PTGs of the control and CKD rats were immediately removed by microsurgery and frozen in liquid nitrogen [8, 17].

### Serum determinations

Serum samples were isolated from blood by centrifugation at 3000 rpm for 5 min at room temperature. Urea nitrogen (UN), creatine (Cr), Ca, Pi, and intact PTH (iPTH) levels were analyzed as previously reported [8]. FGF23 concentration was then measured using intact FGF23 enzyme-linked immunosorbent assay kit (Kainos Laboratories, Tokyo, Japan). 25(OH) D and 1,25(OH)_2_D was measured by outsourcing to SRL (Shinjuku-ku, Tokyo, Japan). The number of animals in the Control NP and Control HP groups was 10 and the other groups had 11 animals.

### Immunohistochemistry

Immunohistochemical staining was performed as described previously [8]. Paraffin-embedded samples were cut into 4-μm sections. Detection of Ki67 was performed, as previously described [8]. Six animals were assigned to each group. We then performed immunostaining using anti-VDR (SC-13133; Santa Cruz Biotechnology Inc., CA, USA) and anti-CaSR (ab19347; Abcam, UK) as the primary antibodies and AlexaFluor 488 and 546 as the secondary antibodies. Before incubation with primary antibodies, the tissue sections were treated with HistoVT one (Nacalai Tesque, Kyoto, Japan) (CaSR, VDR) at 105 °C for 20 min (CaSR, VDR) for antigen retrieval. The sections were then incubated with secondary antibodies at room temperature for 1 h. Each section was examined under a fluorescence microscope (LSM880 confocal; Carl Zeiss, Munich, Germany); three animals were selected for each group.

### RNA isolation and quantitative real-time PCR

Total RNA extraction and first-strand cDNA synthesis were performed as previously described [8]. Six animals from the Control NP, Control HP, CKD NP, and CKD HP groups and seven animals from the other groups were assessed. First-strand cDNA was synthesized from 25 ng of RNA. The TaqMan probe identifiers are listed in Supplemental Table S1.

### Quantitative analysis of DNA methylation using real-time PCR (qAMP)

qAMP was performed as previously reported [8]. The number of animals in the Control group was 6 and in the CKD group was 6. Then 100 ng of each DNA sample was digested with *Hap*II (TaKaRa) and *HhaI* (TaKaRa). All primer sequences used in this study are listed in Supplemental Table S2.

### Statistical analysis

ANOVA followed by Mann–Whitney’s test with Bonferroni correction was used for post-hoc analysis in relation to the biochemical examination, with *p* values of < 0.05 in ANOVA test and 0.0083 in post-hoc analysis considered as statistically significant. Results were presented as mean ± standard deviation (SD).

## Results

### CKD model with SHPT induced with adenine-containing and high-phosphorus diet

Biochemical parameters and body weight (BW) of male adenine-free and adenine-induced rats receiving two types of phosphorus diets are shown in Table 1. The significant increase in UN level was confirmed in the CKD rats compared with the control rats, and no significant difference was found between CKD NP rats and CKD HP rats. Similarly, Cr was also significantly higher in CKD rats than in the control rats; however, a significant decrease was confirmed in CKD HP rats rather than CKD NP rats. BW was significantly lower in the CKD group than in the control group. Although there was no significant difference, the CKD HP group tended to show decrease in BW compared with the CKD NP group. The loss of BW might be the reason why CKD HP had lower Cr levels than the CKD NP group. Namely, there was no significant difference in the severity of CKD status between the CKD groups.

The significant decrease in Ca level and the significant increase in Pi level were confirmed in CKD HP rats compared with the other three groups. iPTH levels in CKD NP rats and CKD HP rats significantly increased about 10 times and 20 times respectively compared with the control groups. Then, active vitamin D, 1,25(OH)_2_D was significantly reduced in the CKD groups compared with the control groups. There was no significant difference between CKD NP rats; however, 1,25(OH)_2_D tended to be lower in the CKD HP rats. Similar to 1,25(OH)_2_D, 25(OH) D was significantly lower in the CKD group than in the control group. FGF23 tended to be higher in the CKD NP group than in the control group; only in the CKD HP group, the level was approximately three times higher than that in the other three groups, which was significant.

**Fig. 1:**
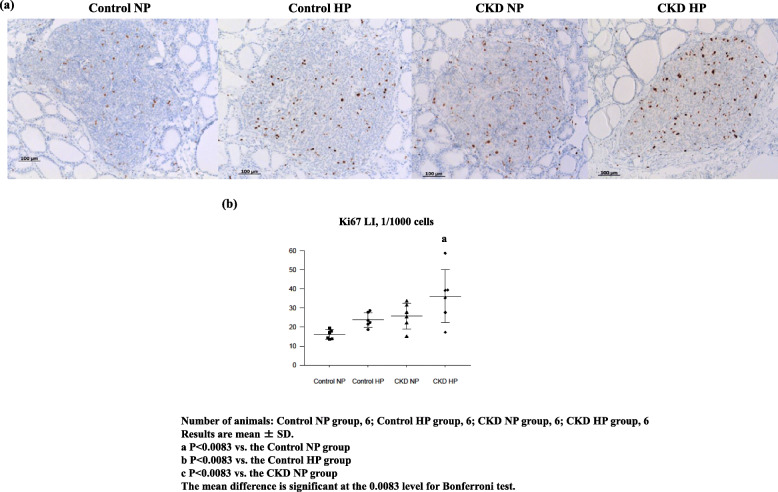
Representative immunohistochemical findings of Ki67 and hematoxylin counterstain (× 100) (**a**), and the differences in the mean labeling index of Ki67 protein expression (**b**) among the four groups: Control NP rats, Control HP rats, CKD NP rats, and CKD HP rats. **a** Nuclear Ki67 expression. Many nuclei in CKD HP were positively stained for the Ki67 protein. **b** In CKD HP rats, Ki67 expression was significantly increased compared with that of the other groups, then the number of Ki67 positive cells tended to increase in the Control NP rats and CKD NP rats, though not significantly different. Number of animals: Control NP, 6; Control HP, 6; CKD NP, 6; and CKD HP, 6. Results are presented as the mean ± SD. The mean difference is significant at the 0.0083 level for Bonferroni test

Next, we analyzed Ki67 expression in the four groups. The increase in Ki67 immunohistochemical expression was observed in Control HP rats, CKD NP rats, and CKD HP rats (Fig. 1a), and Ki67 expression cells was significantly higher only in the CKD HP rats (Fig. 1b). When combining serological data and Ki67 expression results, SHPT status corresponded to only CKD HP rats.

### CaSR reduction was observed in CKD NP rats and CKD HP rats

**Fig. 2:**
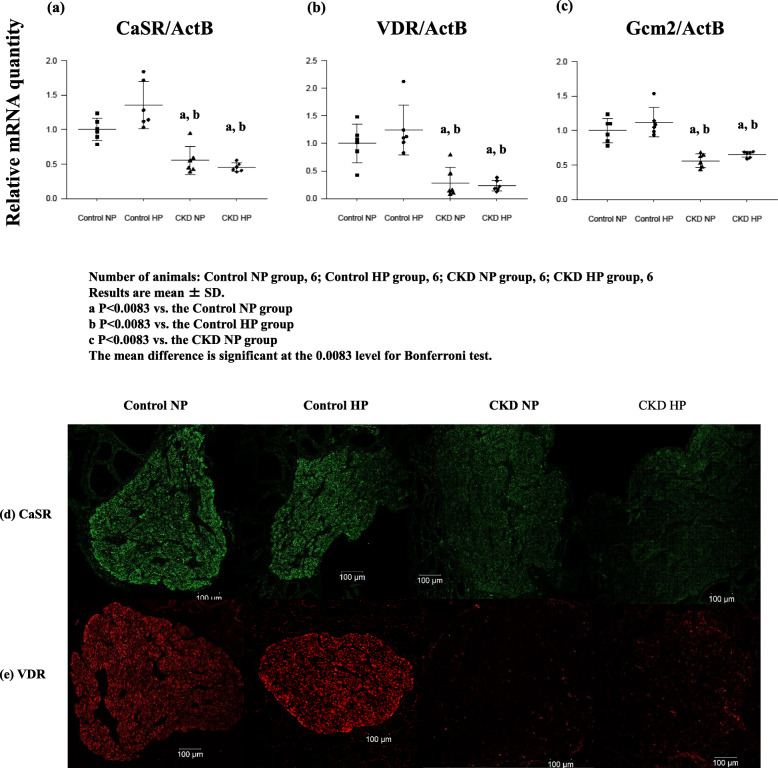
Gene expression (**a**, **b**, **c**) and typical immunohistochemical findings (**d**, **e**) of *CaSR* and *VDR* respectively in PTGs resected from each of four groups: Control NP rats, Control HP rats, CKD NP rats, and CKD HP rats. **a**, **b**, **c***CaSR*, *VDR*, and *Gcm2* RNA levels decreased significantly with or without high phosphorus environment. Gene expression data were measured using real-time RT-PCR and normalized to β-actin. **d**, **e** Representative immunohistochemical findings of *CaSR*, and *VDR* were shown. This result was in agreement with that of gene expression analysis. Number of animals: (**a**, **b**, **c**) Control NP, 6; Control HP, 6; CKD NP, 6; and CKD HP, 6; (**d**, **e**) Control NP, 3; Control HP, 3; CKD NP, 3; and CKD HP, 3. Results are presented as the mean ± SD. The mean difference is significant at the 0.0083 level for Bonferroni test

The change in PTGs *CaSR* mRNA expression and protein expression were quantified using real-time RT-PCR and were analyzed by immunohistochemistry. *CaSR* expression was significantly decreased in CKD groups compared with the control groups (Fig. 2a), furthermore, CaSR protein expression in PTGs were compatible with gene expression assay (Fig. 2d).

### Expressions of VDR, Gcm2, and CaSR

*Gcm2* and *VDR* have response elements in the promoter region of the CaSR gene and upregulate CaSR expression [10, 11]. We previously demonstrated that *VDR* expression significantly decreased only in the CKD HP rat model created 5/6 nephrectomized rats. In this adenine-induced CKD rat model, the expressions of *VDR* in CKD NP rats and CKD HP rats were significantly decreased compared with that in the control groups (Fig. 2b). VDR protein expression in PTGs was compatible with the gene expression assay findings (Fig. 2e); therefore, PTG VDR expression as well as CaSR expression significantly decreased in the presence of a severer CKD environment, which is induced in the short term even without a high phosphorus load. Additionally, *Gcm2* as well as *CaSR* expression in the PTGs significantly decreased in the CKD groups compared with that in the control groups (Fig. 2c). These findings suggest that *CaSR* expression depended on the change in *VDR* and *Gcm2* expressions in CKD.

### Analyses of the expression of various key genes involved in the development of PTGs

To elucidate the reasons for the reduced expression of CaSR, we investigated the transcription factors that are essential for parathyroid development and can influence CaSR expression via Gcm2 expression and have been confirmed the expressions in adult human PTGs [18]. By analyzing at which developmental stage these transcription factors are expressed, it is possible to show indirect upstream and downstream relationships, although the direct causal relationship similar to between *Gcm2* and *CaSR* is unclear.

**Fig. 3:**
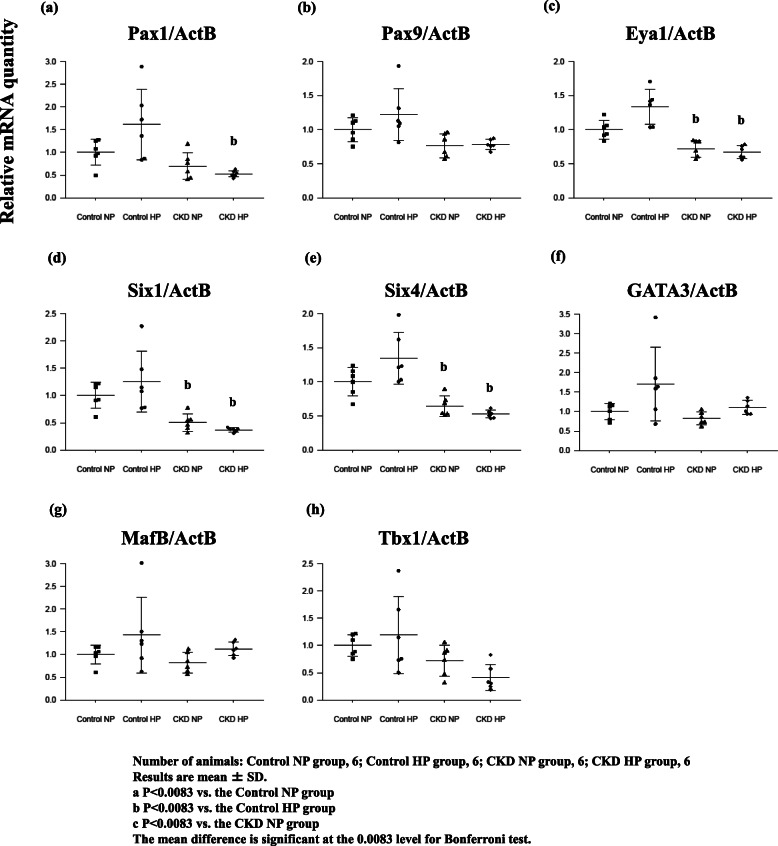
Various key gene expressions concerned with the development of PTGs, *Pax1* (**a**), *Pax9* (**b**), *Eya1* (**c**), *Six1* (**d**), *Six4* (**e**), *Gata3* (**f**), *MafB* (**g**), *Tbx1* (**h**), obtained from rats in each group: Control NP rats, Control HP rats, CKD NP rats, and CKD HP rats. All gene expression tended to increase in Control NP rats; however, there was no significant difference. GATA3 and MafB tended to decrease in the CKD NP rats, but did not show any significant difference. In CKD HP rats, the expressions were almost equivalent to that in the Control NP rats. The expressions of other genes tended to decrease in the CKD NP rats and CKD HP rats; however, there was no significant difference compared with the Control NP rats. Gene expression data was measured using real-time RT-PCR and normalized to β-actin. Number of animals: (**a**) Control NP, 6; Control HP, 6; CKD NP, 6; and CKD HP, 6. Results are presented as the mean ± SD. The mean difference is significant at the 0.0083 level for Bonferroni test

Pax1 and Pax9 expressions tended to decrease in the CKD groups but were not significantly different from the Control NP rats (Fig. 3a, b). Although Eya1 and Six genes, considered to be upstream genes of Pax1 and Pax9 [19], also tended to decrease in CKD rats, there was no significant difference compared with the Control NP group (Fig. 3c, d, and e). *Hoxa3* is expressed in the third and fourth pharyngeal pouch endoderm and neural crest mesenchyme, and inactivation in mice results in the absence of PTG and thymus [20], such as that observed in *Eya1*^−/−^ embryos at E9.5–10.5 [21]. *Foxi1* and *Foxi3*, which are the directed upstream of Gcm2 [22], were barely detectable as was *Hoxa3* expression (data not shown). These findings suggest that *Gcm2* expression did not depend on the change in *Pax1*, *Pax9*, *Eya1*, *Six1*, and *Six4* expressions in CKD.

Moreover, there were no significant differences in the expression of *GATA3*, which is upstream of *Gcm2*, and *MafB*, which activates PTH expression in cooperation with *GATA3* and *Gcm2* although there was an upward trend in Control HP rats (Fig. 3f, g). The expression of *Tbx1*, which changes in the expression of *Pax9* and *Gcm2* expressions [23], tended to decrease in the CKD groups but was not significantly different from that in the Control NP group (Fig. 3h).

### Gcm2 gene expression

**Fig. 4:**
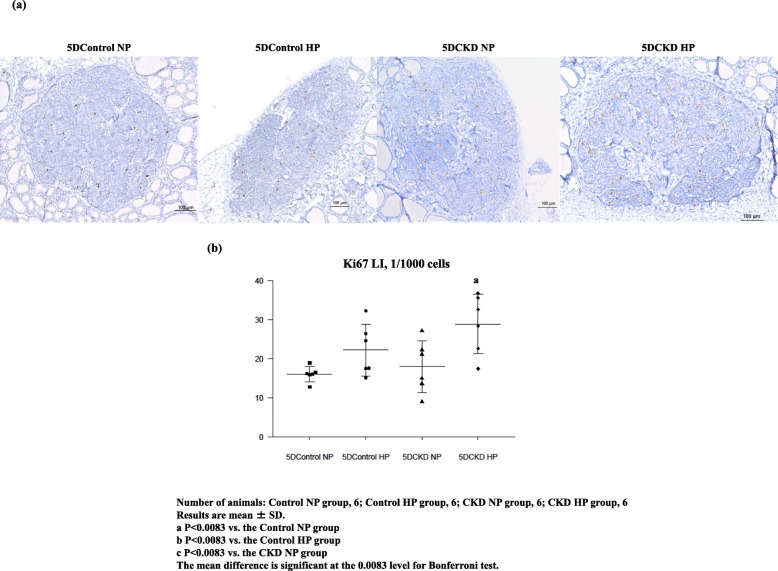
Representative immunohistochemical images of specimens stained with Ki67 and counterstained with hematoxylin (× 100) (**a**) and differences in mean labeling index of Ki67 protein expression (**b**) among 5DControl NP, 5DControl HP, 5DCKD NP and 5DCKD HP groups. **a** Nuclear Ki67 expression. Many nuclei in the CKD HP group were positively stained for Ki67. **b** Ki67 expression significantly increased in the 5DCKD HP group compared with the other groups. Albeit not statistically significant, the number of Ki67-positive cells tended to be higher in the 5DControl HP and 5DCKD NP groups. Number of animals: 5DControl NP, 6;5D Control HP, 6; 5DCKD NP, 6; and 5DCKD HP, 6. Results are presented as mean ± SD. The mean difference is significant at the 0.0083 level for Bonferroni test

**Fig. 5:**
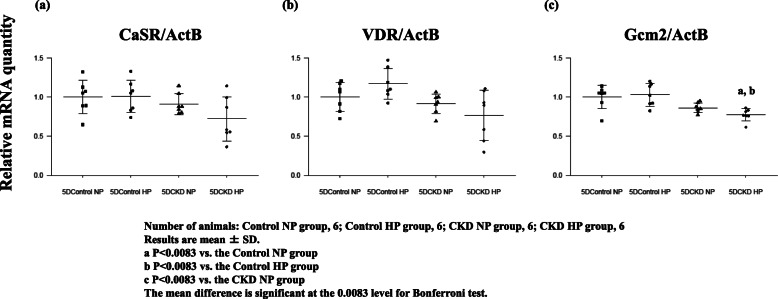
Gene expression of *CaSR* (**a**), *VDR* (**b**), and *Gcm2* (**c**) of PTGs in adenine-induced rats for five days, obtained from rats in each group: 5Dcontrol NP rats, 5Dcontrol HP rats, 5DCKD NP rats, and 5DCKD HP rats. The 5DCKD NP rats and 5DCKD HP rats showed a trend of decrease in the expression of the three genes; however, only *Gcm2* in 5DCKD HP rats had a significant difference compared with 5DControl groups. Gene expression data were measured using real-time RT-PCR and normalized to β-actin. Number of animals: (**a**) 5DControl NP, 6; 5DControl HP, 6; 5DCKD NP, 6; and 5DCKD HP, 6. Results are presented as the mean ± SD. The mean difference is significant at the 0.0083 level for Bonferroni test

To further investigate the role of VDR and Gcm2 genes in detail and their effect on PTG CaSR expression during the development of SHPT, we analyzed data of rats fed a diet containing adenine for five days. The biochemical analysis is shown in Table 2. UN level was significantly elevated in 5DCKD NP rats and 5DCKD HP rats; however, a significant rise in Cr level was only observed in 5DCKD HP rats although Cr showed an upward trend in 5DCKD NP rats. Accordingly, CKD groups showed mild CKD status compared with the control groups, and CKD status in 5DCKD HP rats showed slightly more serious CKD than in 5DCKD NP rats. As for the Ca level, the CKD groups decreased slightly but significantly compared with the control groups, and with respect to the phosphorus level, the CKD rats decreased slightly significantly rather than control groups; however, Ca and Pi were considered to be within the standard values. Regarding PTH, 5Dcontrol HP rats tended shown an increase but there was no significant difference compared with 5Dcontrol NP rats, and the CKD groups showed a significant increase compared with the control groups. Thus, the 5DCKD HP rats showed a significantly higher value even compared with the 5DCKD NP rats. FGF23 concentration was significantly higher in the 5DCKD group than in the 5DControl group, and there was no difference between the 5DCKD NP group and 5DCKD HP group. As for 1,25(OH)_2_D, 5Dcontrol NP rats was significantly elevated as compared with the other three groups, and no significant difference was found in the other three groups. Low levels of 25(OH) D were observed in the 5Dcontrol NP, 5Dcontrol HP, 5DCKD NP, and 5DCKD HP groups with no significant difference observed. Ki67-positive cells were significantly higher only in the 5DCKD HP rats (Fig. 4a, b). We then verified the expression of *CaSR*, *VDR*, and *Gcm2* using the PTGs of these rats. As a result, in the CaSR and VDR, the 5DCKD groups had decreasing tendency compared with the 5Dcontrol groups, but no significant difference was observed (Fig. 5a, b). Then, *Gcm2* expression tended to decrease in the 5DCKD NP rats, but it only decreased significantly in the 5DCKD HP (Fig. 5c). Gcm2 expression in PTGs is significantly decreased at the early stage of CKD even if there is no significant change in CaSR and VDR expression, no decrease in active vitamin D, and levels of Ca and Pi are normal which is a result of significant PTH elevation.

### Hypermethylation of CaSR and VDR in PTGs

Hypermethylation of CaSR and VDR in PTGs were found in 5/6 nephrectomy rat model in which rats were fed a HP diet over 8 weeks. Although there was a significant difference in the degree of hypermethylation, the difference was minor and was insufficient to establish a causal relationship between hypermethylaion and decreased gene expressions [8]. Therefore, we investigated the extent of DNA methylation of CaSR and VDR using quantitative analysis of DNA methylation via real-time PCR (qAMP) in this 2-week adenine-induced rat model. The degree of DNA methylation was low and did not differ significantly among the four groups, as expected (Supplemental Fig. S2). Accordingly, CKD and high phosphorus diet did not lead to the hypermethylation of CaSR and VDR in PTGs. Although there is no CpG island in the promoter region of *Gcm2*, there is one CpG island in the first intron region, i.e., the gene body. The DNA methylation of the first intron was associated with upstream features [24], and we then analyzed the methylation of *Gcm2* CpG island using qAMP. Unfortunately, CpG island of *Gcm2* was not hypermethylated (data not shown).

## Discussion

### PTG CaSR expression in severe CKD

CaSR is a G-protein coupled receptor family member that is most highly expressed in the PTG and renal tubule. CaSR not only plays a pivotal role in mineral homeostasis by regulating PTH and urinary Ca excretion, but also affects noncalcitropic diseases, such as cancer and cardiovascular disease [25]. Although it is not clearly understood which pathway regulates PTH secretion, CaSR activates protein kinase C and inositol 1, 4, 5-triphosphate through G_q/11_, G_i/0_ and G_12/13_, but mainly G_q/11_. Then intracellular Ca increased, and mitogen-activated protein kinase is activated, and PTH secretion is subsequently suppressed [26]. Thus, increases in Ca ion lead to CaSR-mediated suppression of PTH release. In previous reports, hypocalcemia, caused by hyperphosphatemia and reduced 1,25(OH)_2_D in CKD, inactivated CaSR in PTGs and increased PTH secretion [27, 28]. However, in our data, CKD NP rats had significantly decreased 1,25(OH)_2_D, but a significant elevation of iPTH compensated serum Ca and Pi level, namely CKD NP rats in a severe CKD environment with or without high phosphorus load presented a significant decrease in PTG CaSR expression despite maintaining normal serum Ca and Pi levels.

Chang reported that phosphorus overload accelerated CKD progression to end-stage renal disease [29]. In fact, we previously reported that there was a significant difference in the severity of CKD between the high and normal phosphorus diet groups in the 5/6 nephrectomized model rats [8]. Specifically, the UN and Cr levels were 80.9 ± 7.6 mg/dl and 0.66 ± 0.10 mg/dl, respectively, in the 5/6 nephrectomized rats fed a high phosphorus diet; therefore, the rat adenine-induced CKD model used in the present study exhibited severe CKD without a high phosphorus diet. In this adenine-induced rat model, *CaSR* expression significantly decreased in the CKD NP rats, despite not being in SHPT status, as well as in the CKD HP rats. That is, if severe CKD was reached in the short term, PTGs *CaSR* expression significantly decreased even without phosphorus overload.

The pleiotropic effects of VDR are associated with various pathological conditions such as tumorigenesis, immune function, vascular calcification, and lipid metabolism [30]. Under physiological conditions, VDR plays an important role in maintaining mineral homeostasis because it mediates Ca ion reabsorption via the epithelial Ca channel in the distal nephron [31], increases Ca uptake in enterocytes by increasing the expression levels of TRPV5 and TRPV6 [32] and decreasing *PTH* gene transcription [33]. Additionally, VDR has a response element in the promoter region of *CaSR* [11]. Gcm was originally identified in Drosophila as a key regulator of glial cell development [34]. This gene has two mammalian orthologs: *Gcm1* and *Gcm2*. Gcm2 is a transcription factor expressed in the PTH-secreting cells of PTGs and is essential for PTG development [12]. *Gcm2* is expressed in the PTGs throughout human life. If this gene undergoes mutation, the PTGs fail to develop [35]. *Gcm2* expression can upregulate *CaSR* transcription [10] and its expression helps maintain *CaSR* expression levels in parathyroid cells [36]. As expected, *VDR* and *Gcm2* expression as well as *CaSR* expression, in PTGs significantly decreased in the CKD groups compared with the control groups.

Therefore, we consider that 1,25(OH)_2_D and PTG *VDR* expression are very important for the decrease in PTG *CaSR* expression. Furthermore, *Gcm2*, which directly regulates *CaSR* expression, behaved in the same manner as VDR; thus, Gcm2 is important for the suppression of PTG CaSR expression in a CKD environment.

### Effects of decreased PTG *Gcm2* expression on the onset and progression of SHPT

Interestingly, our data suggested that the decrease in PTG CaSR expression, which is important in the development of SHPT, was caused by the early decrease in PTG Gcm2 expression, followed by the effect of 1,25(OH)_2_D reduction, PTG VDR reduction, hypocalcemia and hyperphosphatemia. Therefore, it seems that Gcm2 plays an important role in the development of SHPT. However, we previously showed that even if there is no Gcm2 expression in the PTGs of Gcm2 conditional knockout mice, PTG CaSR expression was observed to decrease slightly [37]. Therefore, it should be noted that CaSR expression is maintained by various factors. Furthermore, we also described the relationship between Gcm2 gene and cell proliferation in PTGs [37]; our data showed the possibility that the decrease of Gcm2 expression in early CKD did not only affect mineral homeostasis but directly affected cell proliferation in PTGs, that is, the development of SHPT itself. Further research is required to clarify the relationship between Gcm2 and SHPT.

### Role of various genes expressed in immature parathyroid cells in adulthood and SHPT

The promoter region of Gcm2 contains the binding sites for Pax1 and Pax9, which are essential for parathyroid development but are not specific to PTGs [38]. Deletion of Pax1 or Pax9 results in failure of the thymus and PTGs [16]. *Eya1* and *Six* are involved in the development of the pharyngeal pouch and head; thus, their absence can result in the malformation of multiple organs including the parathyroid and thyroid glands, kidney, and ear. These genes are considered to be upstream of Pax1 and Pax9 [19]. *GATA3*, a zinc finger enhancer-binding protein, regulates the transcription of numerous genes in vertebrate embryonic development. *GATA3* is upstream of *Gcm2* and causes *Gcm2* dysregulation; its haploinsufficiency causes the hypoparathyroidism, deafness, and renal anomaly syndrome, which results in these three pathologies [39, 40]. Furthermore, there is a report that GATA3 and MafB in association with Gcm2 can transactivate PTH gene expression [41]. *MafB* is a member of the Maf family of bZip transcription factors and plays important roles in the developmental processes of various tissues. *MafB* also regulates PTG separation from the thymus and migration toward the thyroid glands and synergistically activates *PTH* expression following *Gcm2* interaction [42]. These gene expressions were expected to undergo dynamic changes due to CKD environment and high phosphorus diet, but no significant differences were observed. It may be possible to say that these gene expressions were maintained despite the presence of CKD and a high phosphorus environment.

It was very interesting that the genes expressed only in primitive cells such as the Eya gene and Six gene were expressed in adult parathyroid cells, although there was no significant difference in gene expression depending on the whether there was a CKD environment and/or high phosphorus diet. It is unclear what role these genes have in maintaining parathyroid function. Therefore, to analyze the role of these differentiating genes in the adult PTGs is very important from the viewpoint of maintaining normal parathyroid function as well as SHPT. According to our data, these gene expressions were not significantly different between the four groups; however, it may these may be essential.

### Effect of a high phosphorus diet on PTGs

In the present study, the number of Ki67-positive cells significantly increased only in the group fed adenine-containing high phosphorus diet and was not related with the severity of CKD or the period of exposure to the CKD environment and high phosphorus diet. The CKD environment alone tended to accelerate parathyroid cell proliferation, which was significantly augmented only when the CKD environment was loaded with a high phosphorus diet. The 1,25(OH)_2_D, CaSR and VDR expression levels in the PTGs, which suppress parathyroid cell proliferation, were not significantly different between the CKD NP and CKD HP groups; however, FGF23, which also suppresses parathyroid cell proliferation [43, 44], was significantly increased in the CKD HP group. This significant change is consistent with the more severe increase in the PTH levels in the CKD HP group. FGF23 is involved in parathyroid hyperplasia via the NFAT pathway in the absence of Klotho [45]. In our rat model, the PTG *Klotho* expression was not significantly different among the four groups (Supplemental Fig. S3). These results suggest that FGF23 might attempt to suppress the PTGs in the CKD HP group. However, although PTH was significantly elevated in the 5DCKD HP group and not in any of the other three groups, FGF23 was significantly increased in both the 5DCKD NP and 5DCKD HP groups compared with the 5DControl groups, which did not significantly differ from each other. Additionally, the number of Ki67-positive cells significantly increased only in the 5DCKD HP group, suggesting that the early CKD environment and the high phosphorus load led to the parathyroid cell proliferation despite the growth inhibitory effect of FGF23. Our findings indicated that parathyroid cell proliferation occurred in the presence of a high phosphorus diet load even in the early CKD period, during which FGF23 elevation, minimal PTH elevation, and *CaSR* and *VDR* expression in PTGs were not observed. Further, early parathyroid cell proliferation is difficult to predict, at least based on the serological data. Dusso et al. and Cozzolino et al. reported that parathyroid hyperplasia was exacerbated by hyperphosphatemia due to increased expression of transforming growth factor-alpha (TGF-alpha) and epidermal growth factor receptor (EGFR) in the PTGs using the 5/6 nephrectomized rats [46, 47]. However, we did not analyze the expression levels of TGF-alpha and EGFR, which was a limitation of the study. Moreover, further investigation is necessary to determine the cause underlying the lack of a significant increase in FGF23 in the 5DCKD HP group compared with the 5DCKD NP group.

### Epigenetic modification and expression of *CaSR* and *VDR*

Epigenetics is defined as a heritable change in the pattern of gene expression that is mediated by a mechanism specifically not because of alterations in the primary nucleotide sequence. The epigenome dynamically changes in response to intracellular and/or extracellular factors, and the changes in the epigenome affect the pathogenesis and pathophysiology of numerous chronic multifactorial diseases. Multiple reports have correlated hypermethylation with reduced gene expression [48, 49]. In a long-term CKD rat model, we confirmed a slight but significant DNA hypermethylation state of *CaSR* and *VDR*, although this was not the main cause of the decrease in expression [8]. As expected, neither *CaSR* nor *VDR* showed significant DNA hypermethylation in the short-term CKD rat model reported herein. Certainly, the possibility of DNA hypermethylation of *CaSR* and *VDR* in PTGs of long-term hemodialysis patients cannot be denied; however, hypermethylation does not seem to be the main cause of CaSR and VDR gene suppression in SHPT.

We showed the depression of *CaSR*, *VDR* and *Gcm2* expression in PTGs in a severe CKD environment, as well as the decline in *Gcm2* expression from a fairly early stage. However, rat SHPT did not become nodular hyperplasia. Furthermore, Brown et al. identified the areas of diffuse and nodular chief and oxyphil cells in PTGs of human CKD patients. In those areas, the expression levels of CaSR and Gcm2 genes were significantly different [50]. Therefore, further analyses using human PTGs and focusing on the chief and oxyphil cells in the PTGs are required.

## Conclusion

A severe CKD environment reduced CaSR mRNA and protein expressions even in the absence of a high phosphorus diet. Additionally, VDR mRNA and protein level and Gcm2 mRNA level, which are upstream genes of *CaSR*, were also decreased. Because a high phosphorus load causes more severe CKD, measures to reduce high phosphorus loads, particularly, at early stages of CKD, is very important. Furthermore, *Gcm2* suppression occurred at early CKD stages even when no suppression of active vitamin D was observed, and serum Ca and Pi were maintained at normal levels by elevation of PTH, and a significant change of *CaSR* and *VDR* expressions in PTGs was not observed. Consequently, our data suggest that the cause of the decreased PTG *CaSR* expression was the reduction in *VDR* and Gcm2, which may play an essential role in the onset and progression of SHPT. However, further research is needed.

## Supplementary information

**Additional file 1: Figure S1.** Experimental protocol. CKD was induced by 0.75% adenine containing diet. CKD rats and control rats were maintained for 5 days and 2 weeks on diets containing 0.7% phosphorus or 1.3% phosphorus. **Figure S2.** Analysis of the DNA methylation of Gcm2 using qAMP Methylation status in PTGs of the four groups was analyzed using the restriction enzymes HapII (a, c) and HhaI (b, d). Hypermethylation was not observed. Number of animals: Control NP, 6; Control HP, 6; CKD NP, 6; and CKD HP, 6. Results are presented as mean ± SD. The mean difference is significant at the 0.0083 level for Bonferroni test. **Figure S3.** Gene expression of *Klotho* in PTGs resected from the four groups: Control NP rats, Control HP rats, CKD NP rats, and CKD HP rats. There was no significant difference in these groups.

**Additional file 2. Table S1.** Taqman prove assay. **Table S2.** Primer sequences used for quantitative analysis of DNA methylation using realtime PCR (qAMP).

## Data Availability

The datasets used and/or analyzed during the current study are available from the corresponding author on reasonable request.

## Abbreviations

BW: Body weight
Ca: Calcium
CaSR: Calcium sensing receptor
CKD: Chronic kidney disease
Foxi1: Forkhead box i1
Foxi3: Forkhead box i3
EGFR: Epidermal growth factor receptor
ELISA: Enzyme-linked immunosorbent assay
Eya1: Eyes absent homolog 1
GATA3: Trans-acting T-cell-specific transcription factor GATA-3
TGF-alpha: Transforming growth factor-alpha
Gcm2: Glial cells missing 2
HP: High phosphate
Hoxa3: Homeobox A3
MafB: V-maf musculoaponeuroticfibrosarcoma oncogene homologue B
NP: Normal phosphate
Pax1: Paired box 1
Pax9: Paired box 9
PTG: Parathyroid gland
PTH: Parathyroid hormone
Pi: Phosphorus
RT-PCR: Reverse transcription-polymerase chain reaction
SHPT: Secondary hyperparathyroidism
Six1: Sine oculis-related homeobox 1
Six4: Sine oculis-related homeobox 4
SD: Standard deviation
Tbx1: T-box transcription factor 1
UN: Urea nitrogen
VDR: Vitamin D receptor
