# Supplementary material for: Severe chronic kidney disease environment reduced calcium-sensing receptor expression in parathyroid glands of adenine-induced rats even without high phosphorus diet
Source: BMC Nephrol. 2020 Jun 9;21:219. doi: 10.1186/s12882-020-01880-z (PMC7285719; doi:10.1186/s12882-020-01880-z)
Supplement: Supplementary file 1 — Additional file 1: Figure S1. Experimental protocol. CKD was induced by 0.75% adenine containing diet. CKD rats and control rats were maintained for 5 days and 2 weeks on diets containing 0.7% phosphorus or 1.3% phosphorus. Figure S2. Analysis of the DNA methylation of Gcm2 using qAMP Methylation status in PTGs of the four groups was analyzed using the restriction enzymes HapII (a, c) and HhaI (b, d). Hypermethylation was not observed. Number of animals: Control NP, 6; Control HP, 6; CKD NP, 6; and CKD HP, 6. Results are presented as mean ± SD. The mean difference is significant at the 0.0083 level for Bonferroni test. Figure S3. Gene expression of Klotho in PTGs resected from the four groups: Control NP rats, Control HP rats, CKD NP rats, and CKD HP rats. There was no significant difference in these groups. [file 12882_2020_1880_MOESM1_ESM.pptx]

## Slide 1
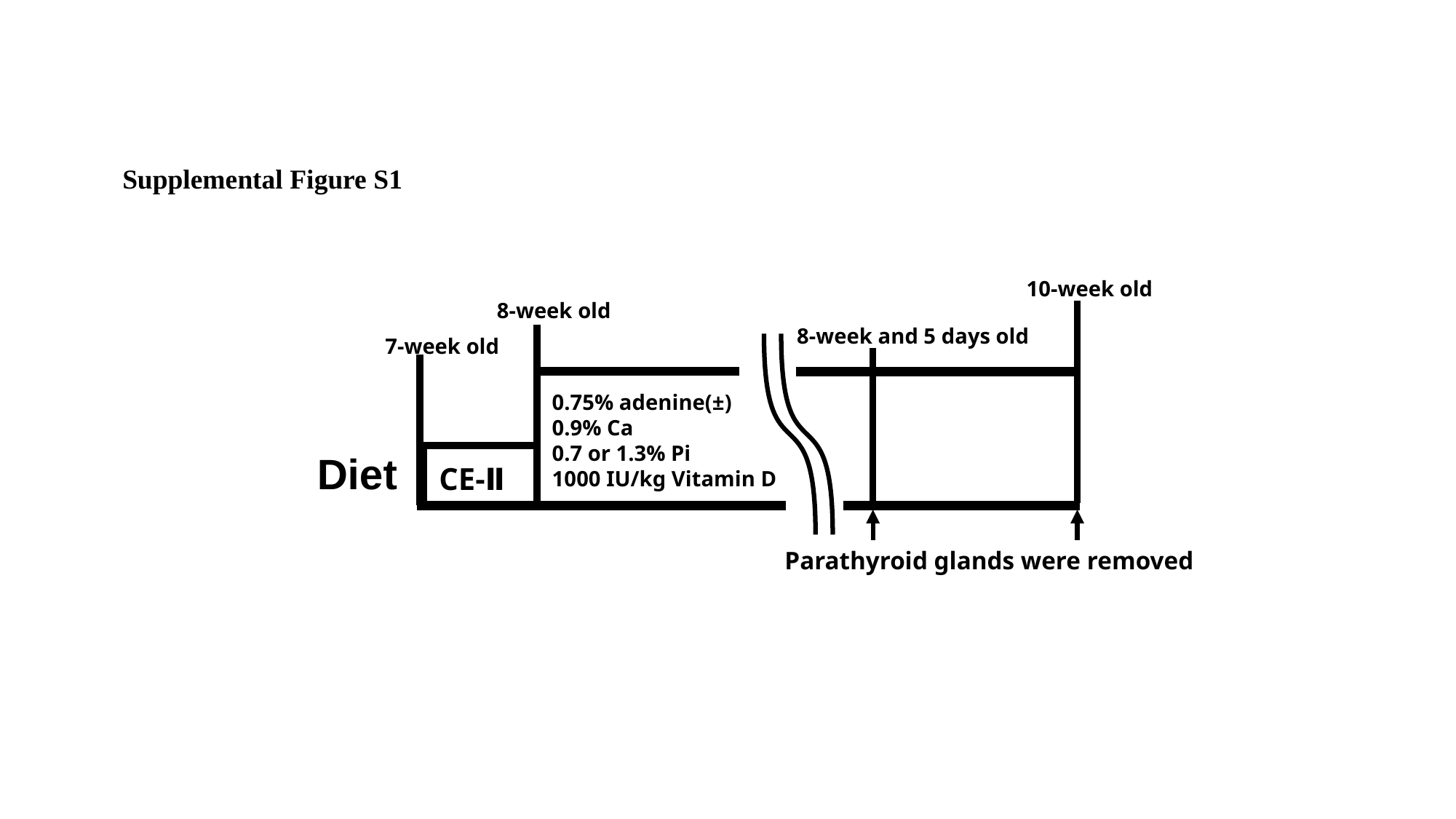

Supplemental Figure S1
10-week old
8-week old
8-week and 5 days old
7-week old
0.75% adenine(±)
0.9% Ca
0.7 or 1.3% Pi
1000 IU/kg Vitamin D
Diet
CE-Ⅱ
Parathyroid glands were removed

## Slide 2
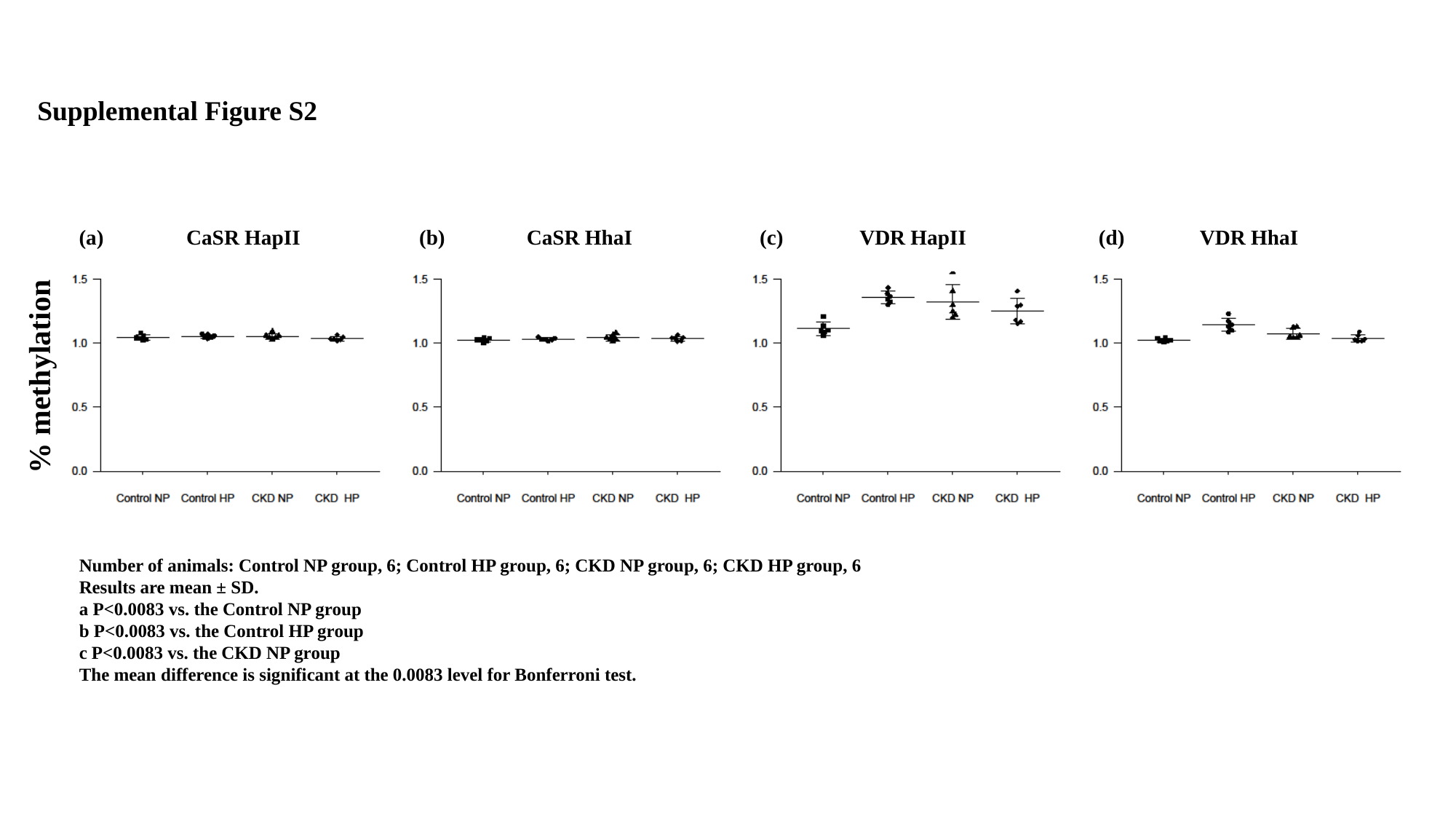

Supplemental Figure S2
(d)
(b)
(c)
(a)
CaSR HapII
VDR HhaI
CaSR HhaI
VDR HapII
% methylation
Number of animals: Control NP group, 6; Control HP group, 6; CKD NP group, 6; CKD HP group, 6
Results are mean ± SD.
a P<0.0083 vs. the Control NP group
b P<0.0083 vs. the Control HP group
c P<0.0083 vs. the CKD NP group
The mean difference is significant at the 0.0083 level for Bonferroni test.

## Slide 3
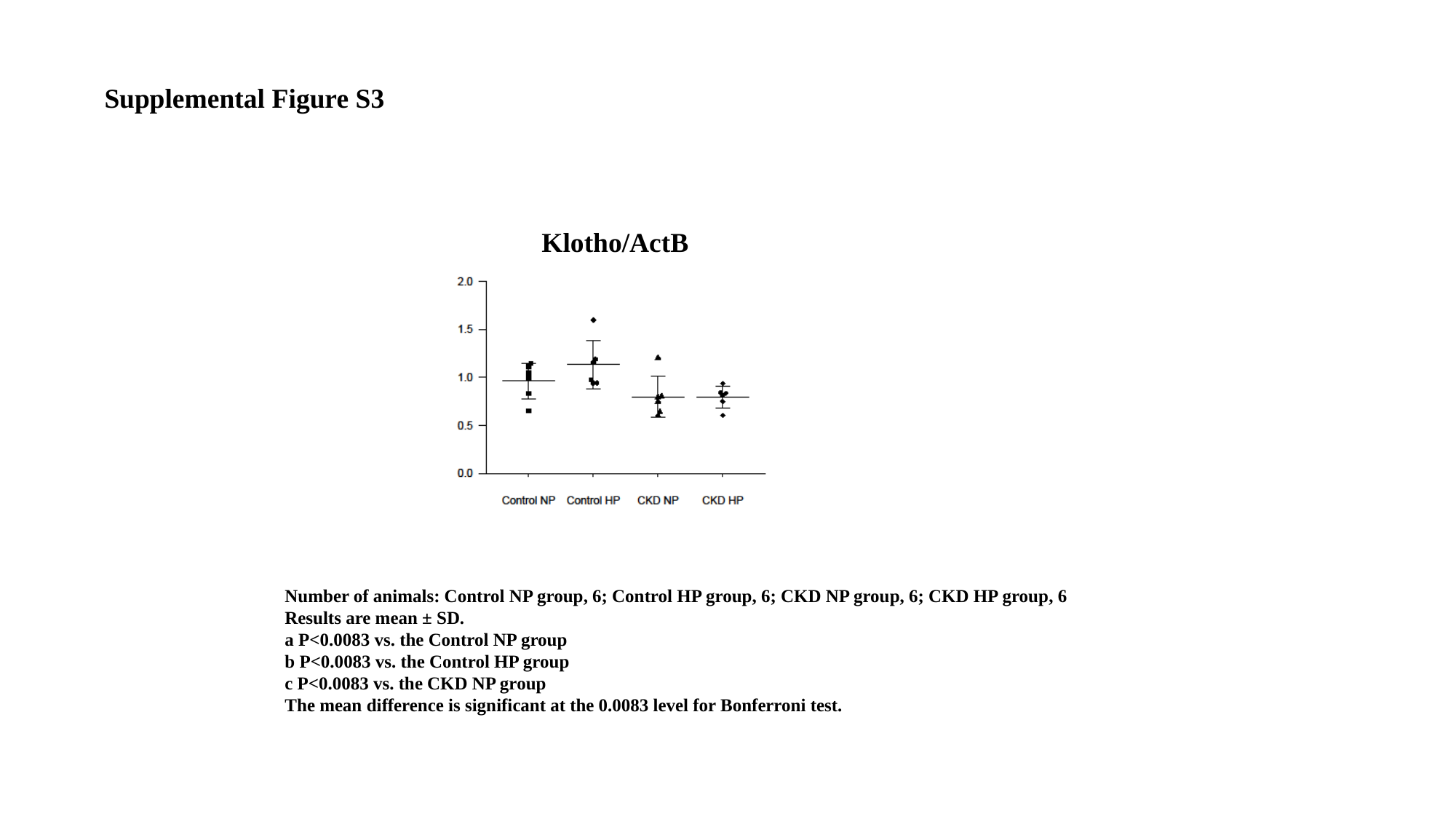

Supplemental Figure S3
Klotho/ActB
Number of animals: Control NP group, 6; Control HP group, 6; CKD NP group, 6; CKD HP group, 6
Results are mean ± SD.
a P<0.0083 vs. the Control NP group
b P<0.0083 vs. the Control HP group
c P<0.0083 vs. the CKD NP group
The mean difference is significant at the 0.0083 level for Bonferroni test.
