# Supplementary material for: Severe chronic kidney disease environment reduced calcium-sensing receptor expression in parathyroid glands of adenine-induced rats even without high phosphorus diet
Source: BMC Nephrol. 2020 Jun 9;21:219. doi: 10.1186/s12882-020-01880-z (PMC7285719; doi:10.1186/s12882-020-01880-z)
Supplement: Supplementary file 2 — Additional file 2. Table S1. Taqman prove assay. Table S2. Primer sequences used for quantitative analysis of DNA methylation using realtime PCR (qAMP). [file 12882_2020_1880_MOESM2_ESM.pptx]

## Slide 1
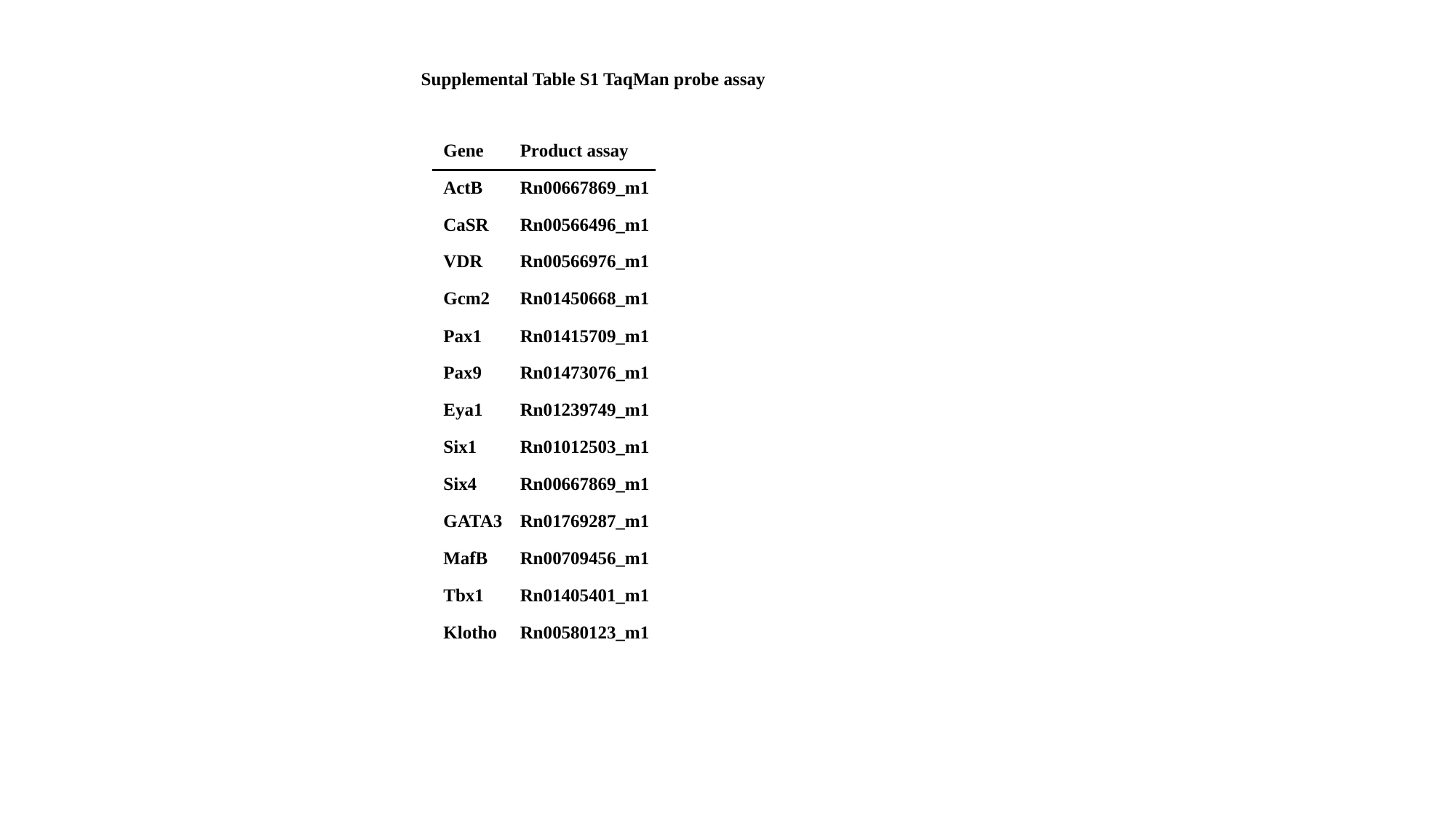

Supplemental Table S1 TaqMan probe assay
| Gene | Product assay |
| --- | --- |
| ActB | Rn00667869\_m1 |
| CaSR | Rn00566496\_m1 |
| VDR | Rn00566976\_m1 |
| Gcm2 | Rn01450668\_m1 |
| Pax1 | Rn01415709\_m1 |
| Pax9 | Rn01473076\_m1 |
| Eya1 | Rn01239749\_m1 |
| Six1 | Rn01012503\_m1 |
| Six4 | Rn00667869\_m1 |
| GATA3 | Rn01769287\_m1 |
| MafB | Rn00709456\_m1 |
| Tbx1 | Rn01405401\_m1 |
| Klotho | Rn00580123\_m1 |

## Slide 2
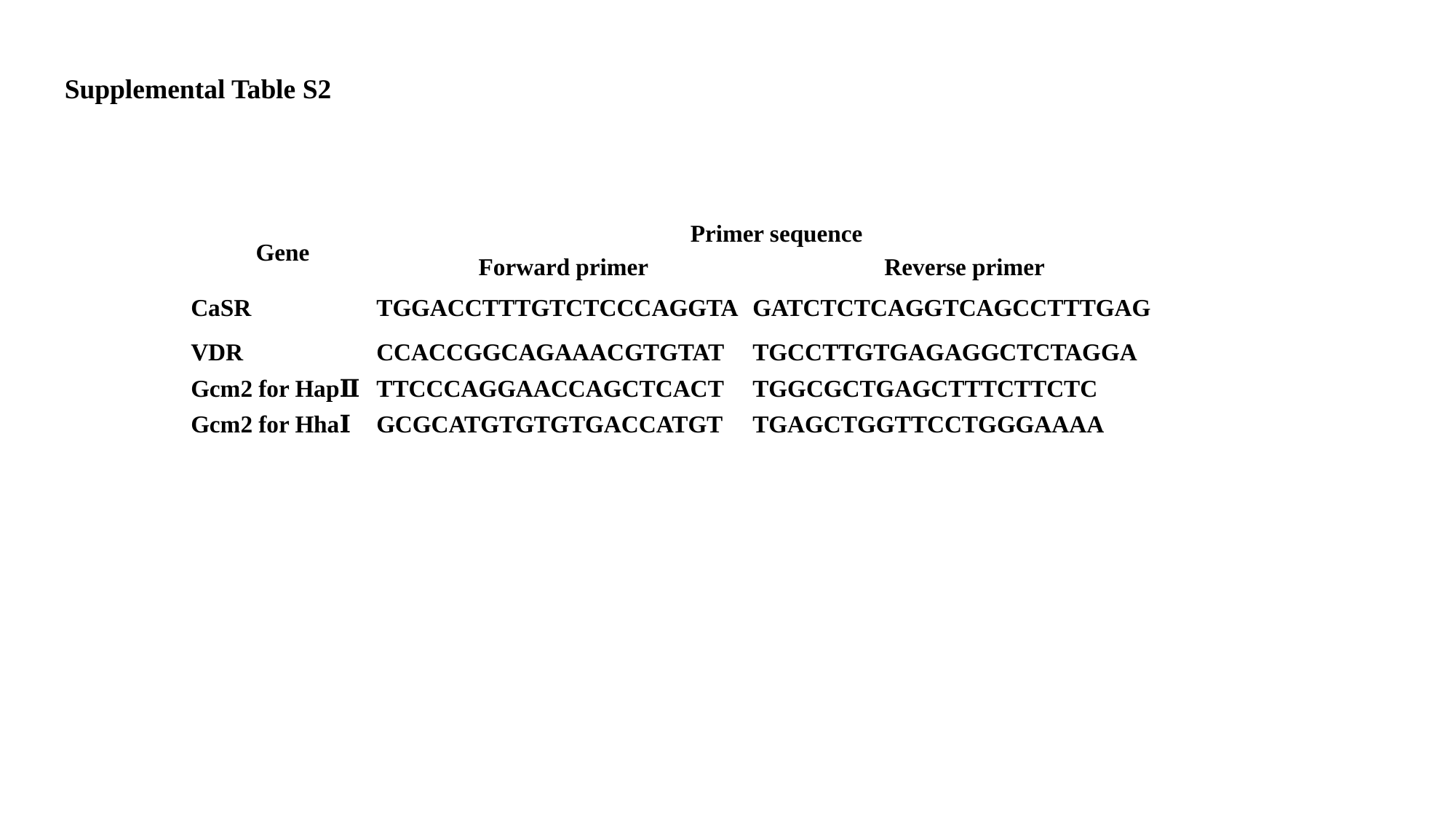

Supplemental Table S2
| Gene | Primer sequence | |
| --- | --- | --- |
| | Forward primer | Reverse primer |
| CaSR | TGGACCTTTGTCTCCCAGGTA | GATCTCTCAGGTCAGCCTTTGAG |
| VDR | CCACCGGCAGAAACGTGTAT | TGCCTTGTGAGAGGCTCTAGGA |
| Gcm2 for HapⅡ | TTCCCAGGAACCAGCTCACT | TGGCGCTGAGCTTTCTTCTC |
| Gcm2 for HhaⅠ | GCGCATGTGTGTGACCATGT | TGAGCTGGTTCCTGGGAAAA |
